# A Lightweight Radar–Camera Fusion Deep Learning Model for Human Activity Recognition

**DOI:** 10.3390/s26030894

**Published:** 2026-01-29

**Authors:** Minkyung Jeon, Sungmin Woo

**Affiliations:** Department of Information and Communication Engineering, Korea University of Technology and Education (KOREATECH), Cheonan-si 31253, Republic of Korea; jun0124z@koreatech.ac.kr

**Keywords:** FMCW radar, human activity recognition, lightweight deep learning model, multimodal sensor fusion, privacy-preserving

## Abstract

Human activity recognition in privacy-sensitive indoor environments requires sensing modalities that remain robust under illumination variation and background clutter while preserving user anonymity. To this end, this study proposes a lightweight radar–camera fusion deep learning model that integrates motion signatures from FMCW radar with coarse spatial cues from ultra-low-resolution camera frames. The radar stream is processed as a Range–Doppler–Time cube, where each frame is flattened and sequentially encoded using a Transformer-based temporal model to capture fine-grained micro-Doppler patterns. The visual stream employs a privacy-preserving 4×5-pixel camera input, from which a temporal sequence of difference frames is extracted and modeled with a dedicated camera Transformer encoder. The two modality-specific feature vectors—each representing the temporal dynamics of motion—are concatenated and passed through a lightweight fully connected classifier to predict human activity categories. A multimodal dataset of synchronized radar cubes and ultra-low-resolution camera sequences across 15 activity classes was constructed for evaluation. Experimental results show that the proposed fusion model achieves 98.74% classification accuracy, significantly outperforming single-modality baselines (single-radar and single-camera). Despite its performance, the entire model requires only 11 million floating-point operations (11 MFLOPs), making it highly efficient for deployment on embedded or edge devices.

## 1. Introduction

Human Activity Recognition (HAR) aims to recognize and classify human activities from various sensing modalities using machine learning algorithms. HAR plays an important role in a wide range of applications including security, rehabilitation and patient monitoring, human–robot interaction, and daily-life behavior analysis such as hazardous activity detection and maintaining regular lifestyle routines [1,2,3,4,5,6,7,8,9].

Activity data can be broadly categorized into image-based data and non-image data. Image-based data include RGB, infrared (IR), and depth images, while non-image data include radar, gyroscopes, and accelerometers. Image-based HAR, which utilizes images captured by cameras, is a traditional and well-established approach [10,11,12]. Because it directly leverages visual information, it can capture fine-grained activity details such as subtle motion changes. However, prior studies have noted that vision-based approaches can raise privacy risks (e.g., potential identity leakage), particularly when high-resolution imagery is used [13]. Motivated by this issue, sensor-based HAR has attracted increasing attention as an alternative, and recent work reports a growing trend toward adopting wearable/sensor-based approaches [14]. Sensor-based HAR [15,16] can capture human motion with fewer privacy-related concerns, but it often exhibits limited discriminability for similar postures compared to image-based approaches.

Radar-based HAR using Frequency-Modulated Continuous Wave (FMCW) radar sensors [17,18,19] is largely robust to environmental factors such as illumination changes and enables contactless motion sensing. Nevertheless, radar signals alone may struggle to accurately discriminate fine-grained body movements or posture variations, which can lead to misclassification among visually similar activities.

**Figure 1:**
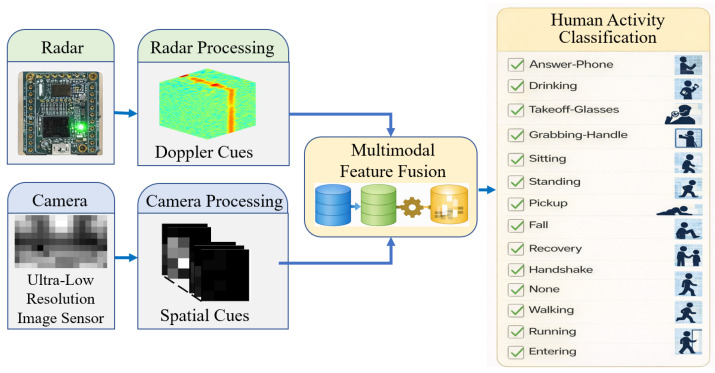
Conceptual overview of the proposed radar–camera fusion framework for privacy-preserving human activity recognition.

As illustrated in Figure 1, this paper proposes a radar–camera fusion-based activity recognition model. Radar data provide range and velocity cues related to motion, while camera data provide complementary spatial cues. However, directly using high-resolution camera images still poses privacy risks due to the possibility of personal identification. Therefore, we assume a 16×9 low-resolution camera stream and use only a 4×5 region of interest; we then compute difference images between consecutive frames to retain only motion-change information. This preprocessing removes appearance and background details, enabling privacy-preserving activity recognition based solely on motion patterns. The proposed model adopts a lightweight architecture for real-time activity recognition and aims to achieve high accuracy without overly complex networks or a large number of parameters. Furthermore, we define 15 daily-life activity classes that cover both common and potentially hazardous behaviors, enhancing practical applicability in real-world environments.

**Contributions.** The main contributions of this work are summarized as follows:

- We propose a privacy-preserving radar–camera HAR framework in which we use only a 4×5 camera stream and form temporal difference representations, retaining motion cues while removing identity-sensitive appearance information.
- We develop a lightweight multimodal fusion model that effectively integrates radar and privacy-preserving camera features, achieving high recognition accuracy with low computational complexity suitable for real-time deployment.
- We conduct comprehensive evaluations on 15 daily-life activity classes, including both static and dynamic motions, and provide detailed analyses (e.g., confusion matrices and modality-attention analysis) to demonstrate the complementary roles of radar and camera.

In this paper, Section 2 reviews the related work, Section 3 describes the proposed methods and presents the experimental results, and Section 4 provides further discussion. Section 5 discusses the limitations of the proposed approach, and Section 6 concludes the paper.

## 2. Related Work

HAR has evolved along two main axes: sensing modalities and model architectures. Sensor-based HAR leverages non-visual signals to enable activity recognition without directly capturing identifiable images, thereby mitigating privacy concerns. Among sensor modalities, FMCW radar has been widely adopted because it is robust to illumination changes and can capture human motion through characteristic signatures in the time, frequency, and range domains. Accordingly, radar-based HAR studies have explored diverse radar representations (e.g., range–Doppler, range–time, and time–frequency maps) together with spatiotemporal learning architectures.

Diraco et al. addressed privacy-sensitive environments by classifying 10 common bathroom activities using micro-Doppler signatures from a MIMO FMCW radar, employing a BiLSTM with multi-head attention to model temporal patterns [20]. Kim and Seo proposed the RD-CNN to learn time–frequency characteristics independently for each range bin [21], while Gianoglio et al. demonstrated real-time operation on embedded hardware using a lightweight CNN–LSTM architecture [22].

Recent research has increasingly focused on enhancing the discriminative power of radar signals through advanced attention mechanisms and efficient feature fusion while maintaining low computational complexity for edge deployment. Li et al. proposed MAEF-Net, which utilizes a multi-attention-enhanced fusion network to capture both local and global time–frequency characteristics from denoised TD maps, ensuring high accuracy with minimal overhead [23]. Similarly, Ding et al. introduced FML-ViT, a lightweight vision transformer that employs cascaded linear self-attention and context broadcasting to significantly reduce the computational complexity inherent in conventional attention-based models [24]. To further resolve ambiguities between similar motion profiles, Yuan et al. developed MFECNet, which fuses range–time (R–T) and Doppler–time (D–T) maps through a lightweight universal inverted bottleneck structure, effectively suppressing noise while enriching the feature representation [25].

Despite these advances in single-sensor processing, radar-only modalities often face inherent limitations in discriminating fine-grained activities with near-identical motion signatures. To overcome such ambiguity, the field has recently shifted towards sophisticated multimodal fusion approaches and the integration of foundation model-based architectures. Lately, the adoption of Multimodal Foundation Models (MFMs) has emerged as a dominant trend, enabling the interpretation of complex human contexts by aligning sensor data with language-based semantics [26,27,28]. These models leverage large-scale pre-training and self-supervised learning to achieve superior generalization across diverse environments.

Furthermore, new paradigms in data fusion and learning have been introduced to address data scarcity and privacy. Recent frameworks have pioneered the use of Federated Learning (FL) for multimodal HAR, integrating heterogeneous data while keeping sensitive information on-device [29]. Concurrently, Modality-Aware Contrastive Learning (MACL) has been investigated to extract robust features from unlabeled multimodal streams, further narrowing the gap between supervised and unsupervised recognition [30].

Multimodal fusion, particularly radar–camera fusion, continues to improve robustness by combining heterogeneous cues. Feng et al. utilized Low-Rank Multimodal Fusion (LMF) to integrate radar RVA maps and camera video sequences [31], and Zhou et al. demonstrated that fusing radar time–frequency spectrograms with high-resolution video significantly improves the discrimination of subtle inter-class differences [32].

Overall, prior work has progressed toward capturing fine-grained motion differences by combining informative radar representations with spatiotemporal models such as CNN–LSTM and Transformers. Multimodal fusion further enhances recognition by integrating radar’s non-visual sensing advantages with the spatial perception capability of cameras. However, many existing fusion approaches, including the latest foundation models, still rely on high-dimensional visual inputs and/or computationally demanding backbones, which can raise privacy concerns and hinder deployment on resource-limited devices. Motivated by these limitations, we propose a lightweight radar–camera fusion framework that emphasizes temporally compact motion representations from an ultra-low-resolution privacy-preserving camera stream while retaining radar’s robust motion sensing capability.

## 3. Proposed Methods

In this study, we propose a radar–camera-based deep learning model for recognizing 15 daily-life activities, including potentially hazardous behaviors. The proposed model improves recognition accuracy by fusing complementary radar and camera cues. To address privacy concerns in camera-based HAR, we use only an ultra-low-resolution camera stream in which personal identification is infeasible, and only motion-change information is preserved. To evaluate the proposed multimodal framework, we collected a dedicated dataset using a synchronized radar–camera sensing setup, as described in the following subsection.

### 3.1. Data Collection

**Figure 2:**
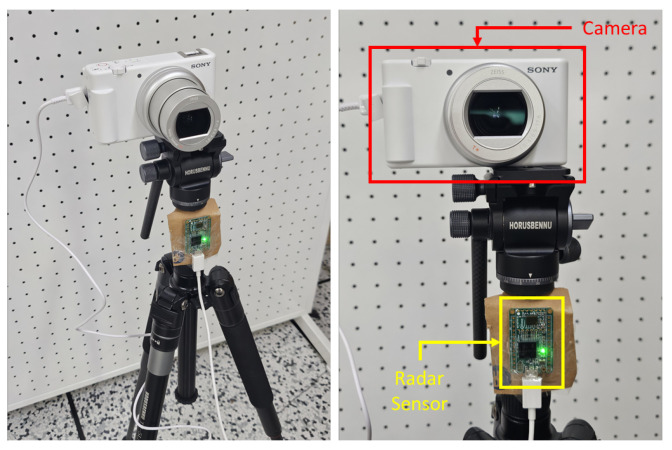
Radar and Camera Hardware Setup.

Multimodal data were collected using an Infineon BGT60TR13C FMCW radar sensor (Infineon Technologies AG, Neubiberg, Germany) and a Sony ZV-1M2 camera (Sony Group Corporation, Minato, Tokyo, Japan), as illustrated in Figure 2. The BGT60TR13C operates at 60 GHz and was configured as summarized in Table 1. The camera captures video at 30 fps with a native resolution of 1280 × 720 pixels. For each recorded sample, approximately 3 s of synchronized radar and camera data were acquired.

All recordings were conducted indoors under normal lighting conditions without introducing artificial illumination changes. To incorporate environmental and viewpoint variability, data were collected from three different viewpoints and under multiple background configurations: (i) a background surface located at approximately 3.5 m from the sensors, (ii) a closer background surface at approximately 2 m, and (iii) a setup in which a doorway was visible to capture subjects entering and exiting the room. These configurations also introduced variation in the subject-to-sensor distance.

The dataset contains 15 daily activities. Detailed descriptions and representative examples of the 15 activities are provided below.

1. *Answer-Phone*: The subject stands in place and raises an arm to the ear, simulating answering a phone.
2. *Drinking*: The subject stands in place and brings a bottle to the mouth, simulating drinking water.
3. *Takeoff-Glasses*: The subject stands in place and removes glasses.
4. *Grabbing-Handle*: The subject stands in place and grasps a door handle.
5. *Sitting*: The subject transitions from a standing position to a seated position.
6. *Standing*: The subject transitions from a seated position to a standing position.
7. *Pickup*: The subject bends down to pick up an object from the floor and then stands up.
8. *Fall*: The subject falls or collapses onto the floor.
9. *Recovery*: The subject raises the upper body from a lying position on the floor.
10. *Handshake*: The subject stands and raises one or both hands, waving them.
11. *None*: The subject remains still in either a sitting or standing posture.
12. *Walking*: The subject walks in various directions and at different speeds.
13. *Running*: The subject runs in various directions and at different speeds.
14. *Entering*: The subject enters the room from outside through the door.
15. *Exiting*: The subject exits the room through the door.

For each activity, 450 scenes were recorded, resulting in a total of 6750 multimodal samples. The dataset includes recordings from two subjects, and both subjects appear in the training and test sets. To increase diversity in spatial conditions, we collected the dataset under multiple subject-to-sensor distances and viewing angles. Specifically, *Answer-Phone*, *Drinking*, *Takeoff-Glasses*, and *Grabbing-Handle* were recorded at two fixed distances (1.5 m and 2 m) with subjects positioned at predefined viewing angles, whereas the remaining activities (*Sitting*, *Standing*, *Pickup*, *Handshake*, *None*, *Fall*, *Recovery*, *Walking*, and *Running*) were recorded across three distance ranges (1.5–2 m, 2–2.5 m, and 2.5–3 m) and a broader set of viewing directions/angles. Table 2 summarizes the dataset acquisition settings.

Radar–camera synchronization was implemented in Python. Each sample contains 2.7 s of radar data (524,288 signal values) and a sequence of 41 camera frames.

**Figure 3:**
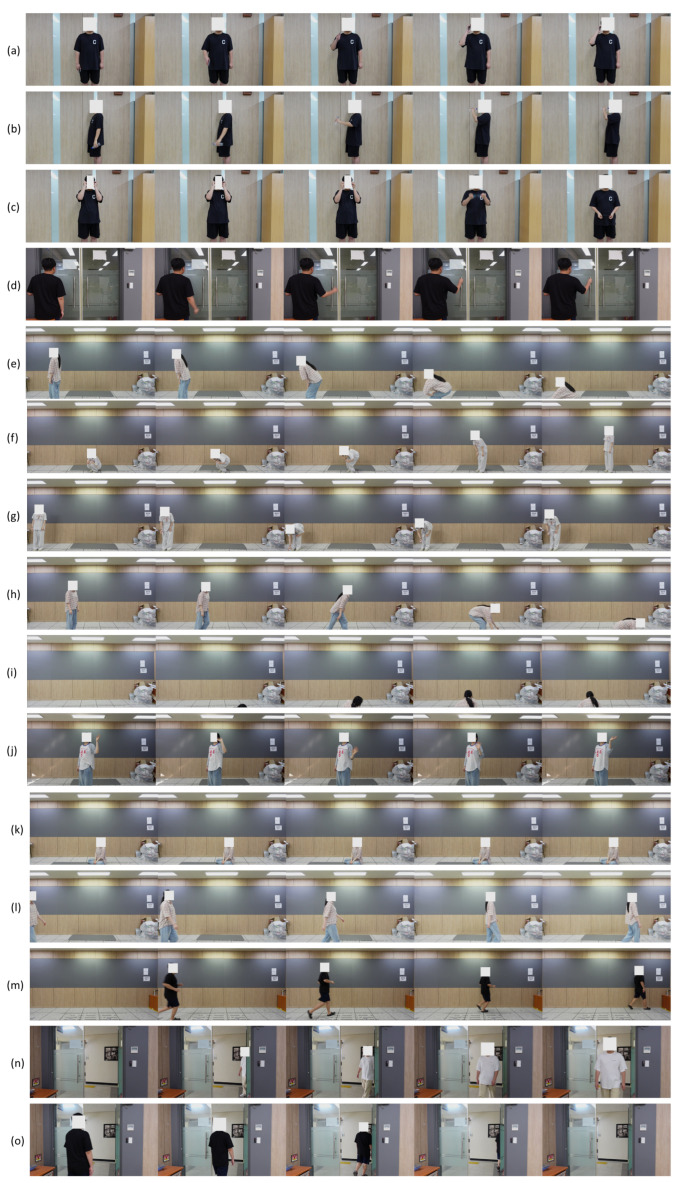
Examples of 15 Human Activities: Five Representative Images in Temporal Order Collected in This Study: (**a**) *Answer-Phone*; (**b**) *Drinking*; (**c**) *Takeoff-Glasses*; (**d**) *Grabbing-Handle*; (**e**) *Sitting*; (**f**) *Standing*; (**g**) *Pickup*; (**h**) *Fall*; (**i**) *Recovery*; (**j**) *Handshake*; (**k**) *None*; (**l**) *Walking*; (**m**) *Running*; (**n**) *Entering*; (**o**) *Exiting*.

Figure 3 presents five representative frames selected from the original 41-frame image sequence for each activity. These examples illustrate the spatiotemporal progression of each activity and highlight that the dataset captures realistic motion patterns and natural continuity of daily human behaviors.

### 3.2. Preprocessing

#### 3.2.1. Radar Data

**Figure 4:**
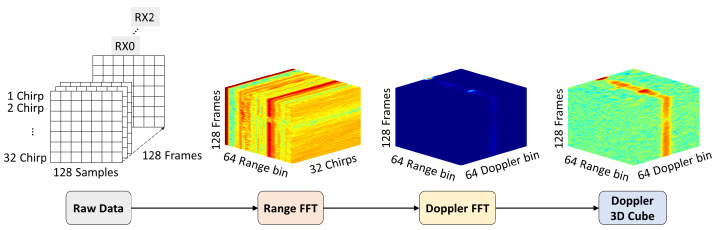
Process of Generating a Doppler 3D Cube from Raw Radar Data.

The raw radar data are FMCW-format floating-point values collected using the IfxRadar SDK, and 128 frames are stored to form a single scene. As shown in the Raw Data stage of Figure 4, each frame consists of 32 chirps, and each chirp contains 128 ADC samples. Consequently, the total data size for one scene comprises 524,288 signal values. At this stage, the raw signal is a floating-point value approximately in the range of −1 to +1. This signal is converted into a 12-bit integer representation, i.e., within the range of 0 to 4095. As a result, a total of 32 chirps×128 samples=4096 samples are generated per frame, and this process is repeated over 128 frames, yielding a total of 524,288 integer values. These data are stored as text files for each receiving antenna of the radar sensor, resulting in three separate radar data text files. In this study, the raw data acquired from the FMCW radar system are transformed into a sequence of Range–Doppler [31,32,33] maps, referred to as a Doppler cube, and used as input to the activity recognition model. The data preprocessing is performed on a per-frame basis following the steps described below.

1. Raw Data: The received frame data were reconstructed into a two-dimensional matrix of size $\left(N_{\mathrm{chirp}},N_{\mathrm{sample}}\right)=\left(32,128\right)$. To improve signal processing accuracy, the mean value of each chirp was removed to compensate for DC offset. In addition, a recursive Moving Target Indicator (MTI) filter was applied to suppress strong clutter components originating from stationary background objects. The MTI filter isolates moving target signals by subtracting the clutter component estimated from the previous frame from the current radar frame. The MTI-filtered output $D_{t}$ is computed by removing the previous clutter estimate $M_{t-1}$ from the current frame $F_{t}$ as follows:
  (1) $$D_{t}=F_{t}-M_{t-1}$$
  The clutter estimate is then updated using a weighted average of the current frame $F_{t}$ and the previous estimate $M_{t-1}$:
  (2) $$M_{t}=\alpha \cdot F_{t}+\left(1-\alpha \right)\cdot M_{t-1}$$
  where $M_{t}$ is the updated clutter estimate, and α is the recursive coefficient controlling the update rate of the clutter model. In this study, α=0.8 was used, indicating that 80% of the clutter estimate is derived from the current frame while the remaining 20% is retained from the previous estimate. As the frame index progresses, the clutter estimate gradually converges, thereby effectively suppressing static components while preserving signals from dynamic targets.
2. Range-FFT: A one-dimensional FFT was performed along the sample direction $N_{\mathrm{sample}}$ on the MTI-processed signal to extract range information. A Blackman–Harris window was applied to reduce sidelobes in the FFT spectrum. Half of the total samples, corresponding to $N_{\mathrm{range}}=\frac{N_{\mathrm{sample}}}{2}=64$ range bins, were retained, and the result was reshaped to a size of $\left(N_{\mathrm{range}},N_{\mathrm{chirp}}\right)=\left(64,32\right)$. The cube obtained after the Range FFT corresponds to the Range FFT stage shown in Figure 4.
3. Doppler-FFT: A one-dimensional FFT was performed along the chirp direction ($N_{\mathrm{chirp}}$) on the Range-FFT output to extract Doppler information. To improve Doppler resolution, zero padding with a length equal to $N_{\mathrm{chirp}}$ was applied, expanding the matrix size to $\left(N_{\mathrm{range}},N_{\mathrm{chirp}}\right)=\left(64,64\right)$ before performing the Doppler FFT. The resulting cube corresponds to the Doppler FFT stage shown in Figure 4.
4. Doppler 3D Cube: After taking the magnitude of the final Range–Doppler map, the result was converted to the dB scale. To maintain consistency in the noise level, the converted values were clipped so that they did not fall below −40dB, i.e., $X_{\mathrm{dB}}=\max\left(X_{\mathrm{dB}},-40\right)$. The (64,64) Range–Doppler maps obtained from each frame were stacked over time to form the final Doppler cube with the size of $\left(N_{\mathrm{range}},N_{\mathrm{doppler}},N_{\mathrm{frame}}\right)=\left(64,64,128\right)$. To reduce computational complexity and enable a lightweight model, the input to the radar network was further downsampled to $\left(N_{\mathrm{range}},N_{\mathrm{doppler}},N_{\mathrm{frame}}\right)=\left(32,64,32\right)$.

#### 3.2.2. Camera Data

The camera data were processed to retain coarse motion cues while reducing appearance and background information. Because a native ultra-low-resolution camera was not available, we captured frames at 1280×720 and immediately resized them to the intended 16×9 resolution, after which all subsequent processing was performed only on the 16×9 stream. The preprocessing pipeline then follows two steps: (1) motion-based region-of-interest (ROI) selection to focus on the most informative motion area, and (2) frame resizing and temporal differencing to generate a compact motion representations. This design supports effective activity recognition while reducing privacy risks by suppressing identity-revealing visual details in the representation used for learning and inference.

**Figure 5:**
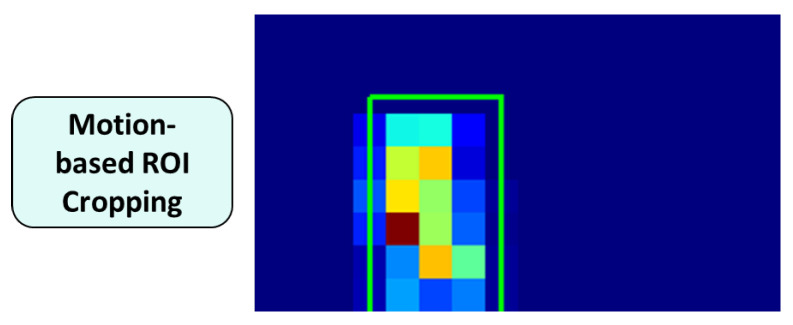
Visualization of motion-based ROI cropping using the SM map defined in Equation (5).

**Figure 6:**
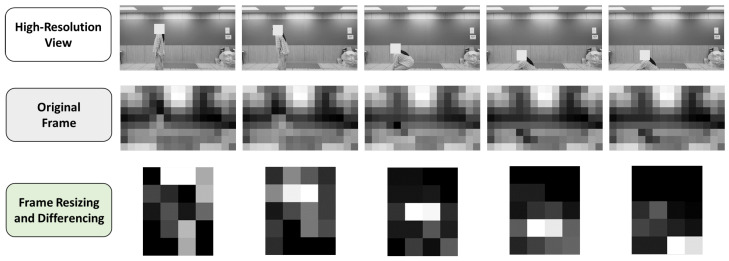
Examples of the original and preprocessed 4 × 5 frames. The high-resolution frames in the first row are shown only for visual comparison.

1. Motion-based ROI Cropping: To localize regions containing significant motion, temporal statistics were computed for each pixel location (x,y) over an image sequence ${\left\{I_{t}\left(x,y\right)\right\}}_{t=1}^{T}$ consisting of *T* frames *at the low-resolution input of 16×9*. The temporal mean $\mu_{T}\left(x,y\right)$ and standard deviation of pixel intensities $\sigma_{T}\left(x,y\right)$ were defined as
  (3) $$\mu_{T}\left(x,y\right)=\frac{1}{T}\sum_{t=1}^{T}I_{t}\left(x,y\right),$$
  (4) $$\sigma_{T}\left(x,y\right)=\sqrt{\frac{1}{T}\sum_{t=1}^{T}{\left(I_{t}\left(x,y\right)-\mu_{T}\left(x,y\right)\right)}^{2}}.$$
  Based on these statistics, a Standard Deviation–Mean ratio map (SM map) was defined as
  (5) $$SM\left(x,y\right)=\frac{\sigma_{T}\left(x,y\right)}{\mu_{T}\left(x,y\right)+\epsilon },\;\epsilon =10^{-6},$$
  where ϵ is a small constant introduced to prevent division by zero. The resulting SM map quantitatively captures relative temporal intensity variations, where higher values highlight regions of frequent inter-frame motion and lower values correspond to the static background. Specifically, the SM map is formulated as the ratio of the temporal standard deviation to the temporal mean (σ/μ) over a window *T*. This coefficient of variation-based approach emphasizes relative temporal fluctuations rather than absolute intensity shifts. This characteristic provides inherent robustness to illumination changes. Sudden lighting variations typically impact the scene globally and simultaneously across most pixels. While such transitions may induce significant inter-frame differences at a specific time step, both the temporal mean (μ) and standard deviation (σ) tend to increase proportionally over the interval *T*. Consequently, since σ and μ in Equation (5) scale in a similar manner, the resulting SM values remain relatively stable. This mathematical property allows the system to effectively distinguish global illumination artifacts from localized, activity-induced motion signatures.
  To reduce noise, a 3×3 mean filter was applied to the SM map, producing a smoothed map $\bar{SM}\left(x,y\right)$. Let $\tau =2\cdot \mathrm{mean}(\bar{SM})$ denote a global threshold computed as twice the average value of the smoothed map. If $\bar{SM}\left(x,y\right)<\tau$, we set SM(x,y)=0, so that regions exhibiting weak temporal variations are suppressed and the subsequent ROI search focuses on motion-dominant areas. An integral image was then computed from the filtered SM map to efficiently evaluate the sum of motion energy within candidate bounding boxes.
  Candidate bounding boxes were generated under the following constraints:
  - The aspect ratio was fixed at *H*:*W* = 2:1 to reflect typical human body proportions.
  - To avoid overly small or overly large boxes in the 16×9 domain, the box height was restricted to H={2,3,5,6,8} pixels, and the width was set to $W=\left\lceil H/2\right\rceil$.
  - For each (H,W) pair, candidate boxes were enumerated by sliding the window over the SM map with a stride of 1 pixel, ensuring dense spatial coverage.
  Among all candidates, the bounding box that maximized the integrated motion energy was selected as the optimal ROI. Consequently, each image sequence was cropped to the smallest region containing the most significant motion, effectively suppressing irrelevant background information while preserving motion-centric features. The selected ROI was shared across all frames within a sequence to ensure temporal consistency. Figure 5 illustrates the motion-based ROI cropping process for a Sitting activity. Regions with higher SM values correspond to areas with significant temporal variations, and the bounding box that maximizes the integrated motion energy is selected as the final ROI.
2. Frame Resizing and Differencing: After ROI cropping, the extracted image sequence was resized to an ultra-low resolution of 4×5 to further remove identity-related visual details while maintaining essential motion patterns. Subsequently, frame-to-frame differencing was applied between consecutive frames to generate a sequence of 40 difference images. Each pixel value in the difference images represents the intensity change between adjacent frames, resulting in large magnitudes in motion regions and near-zero values in static areas. To suppress noise caused by minor illumination variations or sensor artifacts, pixel values with absolute differences less than or equal to 5 were set to zero. Finally, the difference images were normalized based on their absolute values, producing a compact motion representation suitable for real-time processing and privacy-preserving activity recognition. Figure 6 compares an original camera frame with the final preprocessed output. While the original frame contains rich appearance and background information, the preprocessed result retains only coarse motion patterns at an ultra-low resolution, demonstrating effective suppression of identity-sensitive visual details.

Finally, to minimize temporal misalignment between radar and camera data, timestamps from the two sensors were synchronized. Samples exhibiting a time difference greater than 0.05 s were discarded, ensuring temporal consistency between the multimodal inputs.

The processed camera stream is extremely low resolution; nevertheless, it provides crucial cues for action recognition, especially for activities such as Answer-Phone, Drinking, and Takeoff-Glasses, which involve localized movements of specific body parts rather than global body motion.

### 3.3. Multimodal Fusion Framework

**Figure 7:**
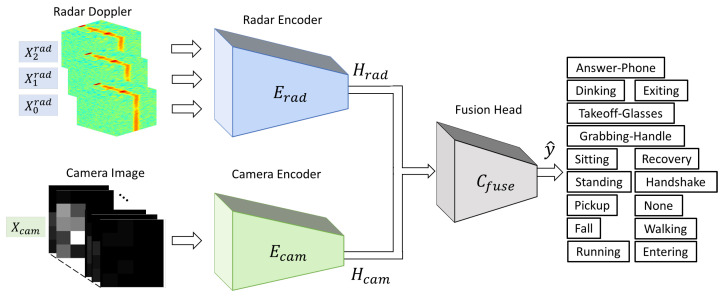
Overall multimodal fusion framework based on modality-specific encoders and classification heads.

Figure 7 illustrates the proposed multimodal fusion framework, which consists of modality-specific encoders and a classification head. The radar encoder and camera encoder independently extract compact temporal representations from their respective inputs, and a classification head is attached depending on the evaluation setting: (i) single-modality baseline evaluation using a modality-specific head, or (ii) multimodal activity recognition using a fusion head.

Let $X_{\mathrm{rad}}=\left\{X_{0}^{\mathrm{rad}},X_{1}^{\mathrm{rad}},X_{2}^{\mathrm{rad}}\right\}$ and $X_{\mathrm{cam}}$ denote the preprocessed radar and camera inputs, respectively, where $X_{i}^{\mathrm{rad}}$ corresponds to the Doppler cube acquired from the *i*-th receiving antenna. Each radar input is independently processed by a shared radar encoder $E_{\mathrm{rad}}\left(\cdot \right)$, and the resulting feature vectors are concatenated to form the aggregated radar representation:

(6) $$H_{\mathrm{rad}}=\mathrm{Concat}\left(E_{\mathrm{rad}}\;\left(X_{0}^{\mathrm{rad}}\right),E_{\mathrm{rad}}\;\left(X_{1}^{\mathrm{rad}}\right),E_{\mathrm{rad}}\;\left(X_{2}^{\mathrm{rad}}\right)\right).$$

Similarly, the camera input is processed by the camera encoder as

(7) $$H_{\mathrm{cam}}=E_{\mathrm{cam}}\left(X_{\mathrm{cam}}\right),$$

where $E_{\mathrm{rad}}\left(\cdot \right)$ and $E_{\mathrm{cam}}\left(\cdot \right)$ represent the radar and camera encoders, and $H_{\mathrm{rad}}$ and $H_{\mathrm{cam}}$ denote the extracted modality-specific feature vectors. To ensure a consistent fusion interface and fair architectural comparisons, all radar and camera encoder variants are configured to output 48-dimensional feature vectors, i.e., $H_{\mathrm{rad}}\in R^{48}$ and $H_{\mathrm{cam}}\in R^{48}$.

For multimodal fusion, the two features are concatenated and classified by a fusion head:

(8) $$\hat{y}=C_{\mathrm{fuse}}\left([H_{\mathrm{rad}};H_{\mathrm{cam}}]\right),$$

where [·;·] denotes feature concatenation and $\hat{y}$ is the predicted activity distribution over the 15 classes. The fusion head is implemented as a lightweight multilayer perceptron (MLP) with nonlinear activation functions and dropout for improved generalization.

In the proposed fusion model, A transformer-based encoder utilizing self-attention is adopted for the radar modality to effectively capture sequential motion patterns. For the camera modality, an LSTM-based encoder is employed to model temporal dynamics in the ultra-low-resolution video stream. In addition, CNN-based encoders employing 3D convolutions are implemented as alternative backbone architectures for comparative analysis, rather than as part of the proposed fusion framework. The detailed architectures of radar and camera encoders are described in Section 3.4 and Section 3.5, and the classification heads are summarized in Section 3.6.

### 3.4. Radar Encoder

This subsection describes the radar encoder $E_{\mathrm{rad}}\left(\cdot \right)$, which extracts a compact temporal feature vector from radar measurements. The radar input is represented as a Range–Doppler–Time spectrum with dimensions (R,D,T)=(32,64,32). Measurements from three receiving antennas are processed by three identical branches, and branch-wise features are aggregated via concatenation to produce the final radar feature vector $H_{\mathrm{rad}}\in R^{48}$.

To analyze the impact of different feature extraction strategies, three radar encoder backbones are considered: (i) a Transformer-based [34,35,36] encoder utilizing self-attention, (ii) an LSTM-based [37,38,39,40] encoder, and (iii) a CNN-based [41,42] encoder employing 3D convolutions. The layer-wise configurations and dimensional transformations of these variants are summarized in Table 3, Table 4 and Table 5.

#### 3.4.1. Transformer-Based Radar Encoder

**Figure 8:**
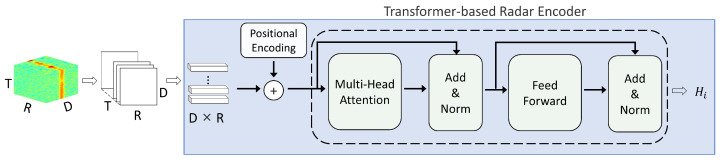
A Radar Model Architecture For A Single Branch (the backbone can be replaced with an LSTM or 3D-CNN).

Transformers provide two key advantages over LSTMs: (i) self-attention can capture global dependencies and complex patterns over long sequences with less information degradation than sequential recurrence [43], and (ii) the architecture enables substantial parallelization, improving training efficiency and scalability on modern hardware [44]. Motivated by these strengths, we employ a Transformer-based radar encoder in the proposed method: the radar cube is rearranged into a temporal sequence, each time step is embedded into a low-dimensional token, and self-attention is applied to model global temporal dependencies. Radar cubes from the three receiving antennas are processed by three independent Transformer encoder branches with identical architectures but separate parameters, enabling branch-wise learning of antenna-specific temporal patterns. A classification (CLS) token and positional embeddings are used, and the CLS output is taken as the branch-wise global representation. The feature extracted from the *i*-th branch is denoted as $H_{i}$ (i∈{1,2,3}). The three branch features are then concatenated to form the radar feature representation $H_{\mathrm{rad}}=\left[H_{1};H_{2};H_{3}\right]$. The detailed architecture and dimensional transformations are summarized in Table 3. Figure 8 illustrates the Transformer-based radar encoder for a single branch, and the corresponding layer-wise structure is summarized in Table 3. Note that the final fully connected (FC) layer shown in Table 3 corresponds to the radar-only classification head used in the single-modality baseline setting; in the fusion framework, the encoder output $H_{i}$ is taken before this classification layer.

#### 3.4.2. LSTM-Based Radar Encoder

In the LSTM-based configuration, the two-dimensional Doppler spectrum at each time step is flattened and fed into a single-layer LSTM. At the beginning of the sequence, the initial cell state $C_{0}$ and hidden state $H_{0}$ are initialized to zero vectors, representing the absence of prior temporal information. The LSTM then iteratively updates its internal states by processing the input sequence ${\left\{x_{t}\right\}}_{t=1}^{T}$, where $x_{t}$ denotes the flattened Doppler spectrum at time step *t*. The hidden state at the final time step $H_{t}$ is used as the representative feature for each antenna branch, and the three branch features are concatenated to form $H_{\mathrm{rad}}$. The corresponding layer-wise structure is summarized in Table 4.

#### 3.4.3. CNN-Based Radar Encoder

The CNN-based radar encoder employs 3D convolutions to preserve the spatiotemporal structure of the radar cube and capture localized motion patterns. Each radar branch produces a compact feature vector through global pooling, and the three branch features are concatenated to form $H_{\mathrm{rad}}$. The layer-wise configuration and dimensional changes are summarized in Table 5.

### 3.5. Camera Encoder

This subsection describes the camera encoder $E_{\mathrm{cam}}\left(\cdot \right)$, which extracts temporal motion features from privacy-preserving camera inputs. The camera input consists of a sequence of preprocessed ultra-low-resolution difference frames with dimensions (H,W,T)=(5,4,40), which retain motion cues while suppressing identity-sensitive appearance information. The camera encoder outputs a 48-dimensional feature vector $H_{\mathrm{cam}}\in R^{48}$.

To enable fair comparisons, three camera encoder backbones are considered: an LSTM-based encoder, a CNN-based encoder employing 3D convolutions, and a Transformer-based encoder utilizing self-attention. The detailed layer-wise configurations are summarized in Table 6, Table 7 and Table 8.

#### 3.5.1. LSTM-Based Camera Encoder

**Figure 9:**
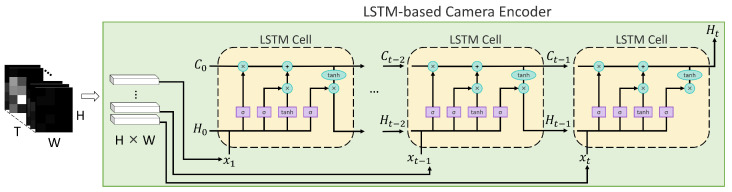
A Camera Model Architecture.

In the LSTM-based configuration, each difference frame is flattened into a compact vector and processed by a single-layer LSTM to model temporal motion patterns across the sequence. At the beginning of the sequence, the initial cell state $C_{0}$ and hidden state $H_{0}$ are initialized to zero vectors, indicating the absence of prior temporal information. The LSTM sequentially updates its internal states by processing the input sequence ${\left\{x_{t}\right\}}_{t=1}^{T}$, where $x_{t}$ denotes the flattened difference frame at time step *t*. The hidden state at the final time step $H_{t}$ serves as the global camera feature vector $H_{\mathrm{cam}}$. Figure 9 illustrates the overall processing pipeline, and Table 6 summarizes the corresponding layer-wise structure. As in the radar case, the final FC layer in Table 6 corresponds to the camera-only classification head used in the single-modality baseline setting; for multimodal fusion, the encoder output $H_{\mathrm{cam}}$ is used prior to this classification layer.

Similarly, the final fully connected layer in Table 6 corresponds to the camera-only classification head used in the single-modality baseline setting; for multimodal fusion, the camera encoder output $H_{\mathrm{cam}}$ is used prior to this layer.

#### 3.5.2. CNN-Based Camera Encoder

The CNN-based camera encoder uses 3D convolutions to capture localized spatiotemporal motion cues from the camera cube and compresses the input into a compact representation via global pooling. The resulting feature vector is used as $H_{\mathrm{cam}}$, and the layer-wise architecture is summarized in Table 7.

#### 3.5.3. Transformer-Based Camera Encoder

The Transformer-based camera encoder treats each frame as a temporal token and applies self-attention to model long-range temporal dependencies in motion patterns. With positional embeddings and a CLS token, the CLS output provides the global representation $H_{\mathrm{cam}}$. The detailed architecture is summarized in Table 8.

### 3.6. Classification Heads

To decouple feature extraction from classification and to support both single-modality baselines and multimodal fusion within a unified framework, different classification heads are attached on top of the encoder outputs depending on the evaluation setting.

For single-modality baselines, a modality-specific head is applied to each encoder output:

(9) $${\hat{y}}_{\mathrm{rad}}=C_{\mathrm{rad}}\left(H_{\mathrm{rad}}\right),\;{\hat{y}}_{\mathrm{cam}}=C_{\mathrm{cam}}\left(H_{\mathrm{cam}}\right),$$

where $C_{\mathrm{rad}}\left(\cdot \right)$ and $C_{\mathrm{cam}}\left(\cdot \right)$ are implemented as a lightweight fully connected layer.

For multimodal fusion, the fusion head $C_{\mathrm{fuse}}\left(\cdot \right)$ takes the concatenated feature vector $\left[H_{\mathrm{rad}};H_{\mathrm{cam}}\right]\in R^{96}$ and performs nonlinear transformations via an MLP before producing the final class prediction. The detailed configurations are summarized in Table 9.

### 3.7. Training Environment

All experiments were conducted using PyTorch 2.5.1 with CUDA 12.4 on an NVIDIA GeForce RTX 4090 GPU. Model training was performed in a Python 3.11.5 environment, and both training and evaluation codes were executed on a Windows 11-based system.

The models were optimized using the Adam optimizer with an initial learning rate of $10^{-3}$ and default momentum parameters $\left(\beta_{1}=0.9,\beta_{2}=0.999\right)$. A cross-entropy loss function with label smoothing was employed to promote stable learning of decision boundaries between activity classes. The batch size was set to 32, and all models were trained for 1000 epochs. To improve optimization stability and convergence, a ReduceLROnPlateau learning rate scheduler was applied based on the validation loss. The learning rate was reduced by a factor of 0.3 if no improvement was observed for 50 consecutive epochs, with a minimum learning rate of $10^{-5}$.

To ensure reproducibility, all experiments were conducted with fixed random seeds for Python, NumPy, and PyTorch. Deterministic behavior was enforced by disabling cuDNN benchmarking and enabling deterministic computation. In addition, gradient clipping with a maximum $\ell_{2}$ norm of 2.0 was applied to prevent gradient explosion during recurrent model training. All models were trained using the same hyperparameter configuration to ensure fair comparisons across different modality combinations. Model selection was performed based on the highest validation accuracy, and the corresponding weights were saved for final evaluation on the test set. A summary of the training environment and key hyperparameters is provided in Table 10, and the evaluation results are reported using accuracy metrics and confusion matrix analyses in the following section.

### 3.8. Dataset Split and Evaluation Protocol

For each activity class, 450 independently recorded scenes were collected and randomly split into training and test sets with a ratio of 5:1. The train–test split was performed randomly; however, to prevent distance-related bias, the test set was constructed by maintaining an equal proportion of samples from each distance condition. The dataset includes recordings from two subjects, and both subjects are present in both the training and test sets. As a result, cross-subject or cross-session validation was not conducted in this study. Instead, the evaluation focuses on assessing the effectiveness of multimodal fusion under consistent subject conditions.

### 3.9. Quantitative Evaluation

To objectively evaluate the proposed approach, we measured classification accuracy as well as computational cost in terms of FLOPs and the number of trainable parameters (Params). These metrics enable an analysis of the trade-off between recognition performance and computational efficiency. Table 11 compares the accuracy, computational complexity, and parameter counts of the single-modality baselines and the multimodal fusion model.

As shown in Table 11, the proposed fusion model achieved a mean accuracy of 98.74% (98.74 ± 0.55%) across five random seeds, outperforming the radar-only and camera-only baselines by 3.48 and 3.11 percentage points, respectively. This improvement indicates that jointly leveraging radar and camera cues yields more discriminative representations than using a single sensor alone. As discussed later, the fusion model particularly enhances recognition of activities that are difficult to distinguish with radar alone due to subtle motion patterns (e.g., *Answer-Phone*, *Drinking*, and *Takeoff-Glasses*), while also improving performance on more dynamic activities such as *Running* and *Entering*. Moreover, the low standard deviation across runs demonstrates that the proposed method is not only accurate but also statistically stable.

In addition, the proposed fusion strategy is benchmarked against Zhou et al. [24]’s CNN–LSTM data-level fusion and Feng et al. [23]’s low-rank multimodal fusion (LMF) under the same dataset and preprocessing pipeline, so that only the network architectures differ across methods. For Zhou et al. [24], radar and camera streams are concatenated at the input, forming a four-channel tensor (three radar channels + one camera channel) for 2D CNN processing; the radar temporal length is aligned to the camera sequence and unified to 40 frames for the subsequent LSTM. For Feng et al. [23], the domain discriminator is removed to isolate the fusion mechanism, and max pooling in the camera branch is applied conservatively to preserve spatial information under the ultra-low-resolution input.

As summarized in Table 11, Zhou et al. [24] and Feng et al. [23] achieve higher accuracies (99.70% and 98.89%, respectively) than the proposed method (98.74%). However, the proposed fusion model requires only 11 MFLOPs and runs in 0.64 ms per inference (approximately 1563 FPS), whereas Zhou et al. [24] and Feng et al. [23] require 2464 and 25,856 MFLOPs, respectively (i.e., ∼224× and ∼2350× higher computational costs). Overall, these results highlight a competitive accuracy–efficiency trade-off suitable for lightweight real-time deployment.

### 3.10. Model Variants: Encoder Backbones and Radar Representations

In this subsection, we compare model variants to analyze the impact of encoder backbones and radar input representations on both single-modality baselines and multimodal fusion performance. Three encoder architectures are considered for both modalities: LSTM-, 3D-CNN-, and Transformer-based encoders. The 3D-CNN encoders apply 3D convolutions to capture localized spatiotemporal motion patterns, whereas Transformer encoders leverage self-attention to model global dependencies. LSTM encoders are well suited for sequential modeling by compressing temporal dependencies into compact feature representations.

We additionally evaluate two radar preprocessing strategies using the same raw radar recordings: a Doppler-cube representation and a Short-Time Fourier Transform (STFT)-based spectrogram representation [45,46]. Unlike the Doppler-based method in Section 3.2.1, the STFT-based approach first performs an FFT for each chirp to obtain range information and then applies STFT along the time axis, yielding a more continuous description of spectral evolution over time.

Table 12 reports the accuracy and computational costs (FLOPs and parameter counts) of the three encoder backbones for radar-only and camera-only settings under identical training conditions. While both radar representations can be used with temporal encoders, the STFT-based representation provides finer temporal continuity, which can benefit sequence models such as LSTMs; this tendency is reflected by the improved radar LSTM performance with STFT compared to the Doppler-based representation.

We further examine how the encoder choices for each modality affect multimodal fusion. Table 13 summarizes the fusion accuracy and computational costs for different combinations of radar and camera encoders under Doppler and STFT radar representations. Across all evaluated combinations, fusion models outperform their corresponding single-modality baselines, confirming that radar and camera provide complementary cues. The best-performing configuration reaches 99.41% accuracy using a Doppler-based radar CNN and a camera LSTM.

### 3.11. Confusion Matrix

We computed confusion matrices to analyze which activity classes benefit most from the proposed fusion approach compared to single-modality classification. We focus on the LSTM-based results for the radar-only, camera-only, and fusion models, where the benefits of fusion are most pronounced. Table 14, Table 15 and Table 16 report the confusion matrices on the test set for the radar-only, camera-only, and fusion models, respectively.

A clear trend is observed for activities dominated by localized or subtle body-part motion, such as *Answer-Phone*, *Drinking*, and *Takeoff-Glasses*. In the radar-only model (Table 14), these classes are frequently confused with one another: *Answer-Phone* is misclassified as *Drinking* 16 times and as *Takeoff-Glasses* 7 times (67/90 correct), while *Drinking* and *Takeoff-Glasses* also show noticeable cross-confusions (77/90 and 73/90 correct). This behavior is consistent with the fact that radar signatures of such actions often exhibit limited range displacement and similar micro-Doppler patterns. By contrast, the camera-only model (Table 15) provides stronger separation for these classes even under privacy-preserving inputs, achieving 85/90 for *Answer-Phone* and 89/90 for *Takeoff-Glasses*. When both modalities are fused (Table 16), the remaining ambiguity is further reduced, yielding 88/90, 84/90, and 90/90 for *Answer-Phone*, *Drinking*, and *Takeoff-Glasses*, respectively.

A second representative failure mode of the radar-only model appears in posture-transition activities, especially *Standing* and *Pickup*. Although both actions involve prominent motion, their dominant component is largely vertical and can produce similar range–velocity trends, leading to severe mutual confusion in Table 14: only 38/90 samples of *Standing* are correctly classified, with 20 misclassified as *Sitting* and 20 as *Pickup*. Likewise, *Pickup* achieves 56/90 correct, with errors distributed across *Standing* (11) and *Handshake* (14). In contrast, the camera-only model maintains high recognition for these classes (89/90 for *Standing* and 88/90 for *Pickup*), suggesting that even ultra-low-resolution motion cues preserve discriminative spatial–temporal changes during posture transitions. Importantly, fusing radar and camera features substantially resolves the radar-only ambiguities, improving *Standing* from 38/90 to 87/90 and maintaining *Pickup* at 88/90 with only a small residual confusion to *Handshake* (2 cases).

To further understand the radar-only failures, Table 17 breaks down misclassifications of *Standing* and *Pickup* by subject-to-sensor distance and viewing angle. The distribution of errors suggests a clearer dependence on distance than on viewing angle: for *Standing*, misclassifications are most frequent at 2–2.5 m (23 cases), compared to 1.5–2 m (16) and 2.5–3 m (13); for *Pickup*, a similar peak is observed at 2–2.5 m (19 cases), compared to 1.5–2 m (9) and 2.5–3 m (6). Overall, these results indicate that radar-only discrimination between posture-transition activities tends to be more challenging at intermediate distances in our setup, while angle-dependent degradation appears less consistent.

Overall, these confusion-matrix analyses confirm that the two modalities contribute complementary strengths: the camera branch is particularly effective for localized or posture-related cues (e.g., *Answer-Phone*, *Takeoff-Glasses*, *Standing*), while the radar branch remains reliable for large-scale dynamic motions (e.g., *Running*, *Entering/Exiting*). The proposed fusion model leverages this complementarity to reduce class-specific confusions and produce more stable separation across both subtle and dynamic activities.

## 4. Discussion

### Class-WiseTransformer Attention Analysis for Modality Contribution

To analyze modality contributions in the radar–camera fusion framework, we design an attention-inspection model in which the *fusion module* is implemented as a Transformer. This design enables explicit visualization of attention weight maps over modality-specific tokens, allowing us to examine how the fusion module attends to radar and camera information for each activity class.

In the analysis model, both the radar and camera feature extractors are implemented using 3D convolutional encoders. Their outputs are tokenized and concatenated to form the Transformer input sequence, and the Transformer produces the final activity prediction. To isolate the behavior of the fusion module during attention analysis, the radar and camera encoders are initialized with pretrained weights obtained from the single-modality 3D-CNN variants trained in Section 3.10 and then kept fixed during the fusion training stage.

**Figure 10:**
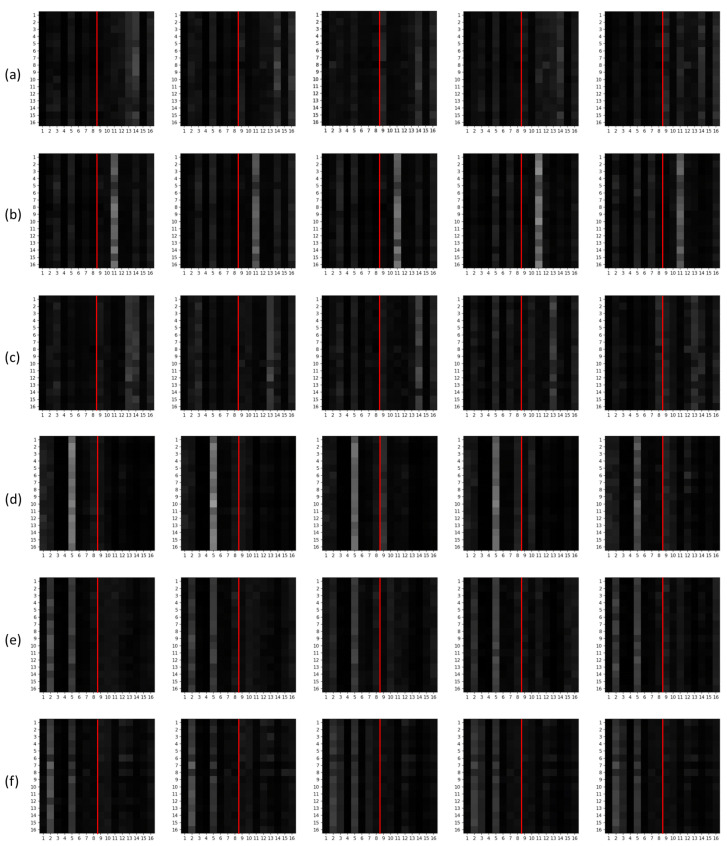
Example class-wise attention maps in the Transformer-based fusion model (tokens 1–8: radar; tokens 9–16: camera): (**a**) Answer-Phone, (**b**) Drinking, (**c**) Takeoff-Glasses, (**d**) Running, (**e**) Entering, and (**f**) Exiting.

We visualize the attention weight maps within the Transformer encoder to investigate how the model distributes attention across radar and camera tokens. The Transformer input consists of a total of 16 tokens, where tokens 1–8 correspond to radar features and tokens 9–16 correspond to camera features, following the tokenization scheme induced by the 3D convolution-based encoders. As these tokens pass through the Transformer encoder, distinct attention distributions emerge depending on the activity class. Figure 10 shows representative attention maps for six activity classes.

In the attention maps, the y-axis denotes the query tokens and the *x*-axis denotes the key tokens. For example, a brighter 10th column indicates that many queries strongly attend to the 10th token, suggesting that the token is informative for classification. To clearly distinguish between modalities, a red boundary line is drawn between radar tokens (1–8) and camera tokens (9–16).

The attention maps indicate that for low-dynamics activities such as Answer-Phone, Drinking, and Takeoff-Glasses, the model tends to assign higher attention to camera tokens. This suggests that ultra-low-resolution difference frames still preserve discriminative cues for subtle arm and upper-body motions, even when overall translational movement is limited. In contrast, for activities involving large-scale motion (e.g., Running, Entering, and Exiting), attention is more concentrated on radar tokens, consistent with radar’s ability to encode time-varying range–Doppler patterns associated with rapid velocity changes and translational movements.

Table 18 reports the average attention ratio assigned to radar and camera tokens for each activity class. Among the 15 activities, Walking, Running, Entering, and Exiting involve stronger translational motion across space. Consistent with the qualitative attention maps, radar tokens receive higher attention weights for these dynamic activities, whereas camera tokens receive higher attention weights for many activities performed mostly in place. Walking exhibits a near-balanced attention ratio, suggesting that both modalities contribute comparably for this class.

## 5. Limitation and Future Work

Although the proposed method demonstrates that even ultra-compact spatial cues from an extremely low-resolution camera can complement neural features and improve action recognition, several considerations are necessary before deploying it in real-world applications. First, our experiments were conducted with only two participants in a single classroom environment. While we additionally collected data at multiple subject–sensor distances, recognition performance may still vary with user-dependent factors such as body size, appearance (e.g., clothing color), and distance to the sensors. Moreover, radar measurements can be affected by environmental factors and electromagnetic interference from nearby electronic devices, which may influence performance.

We also note potential failure cases related to the camera modality. Since the camera branch relies on motion-change cues derived from frame differencing, frequent or abrupt illumination changes (e.g., flickering lights or rapid sunlight variations) may introduce spurious intensity differences and degrade recognition performance. In future work, we plan to improve robustness under such conditions through illumination-robust preprocessing and data augmentation (e.g., brightness/contrast jitter) and by exploring alternative motion representations less sensitive to lighting variations.

More broadly, the generalizability of the proposed approach across different indoor environments, sensor placements, and user populations remains an open question: variations in room layout, furniture configuration, sensor mounting height, and user physical characteristics can alter both radar reflections and low-resolution camera representations. Therefore, expanding the dataset to include multiple environments and a more diverse participant pool is an important next step toward improving real-world applicability.

Finally, although the proposed camera preprocessing is intended to remove identifiable visual cues, this study does not include a quantitative privacy assessment (e.g., re-identification attacks or privacy metrics). Future work will incorporate such evaluations to measure identity leakage directly.

## 6. Conclusions

This study proposed a lightweight radar–camera fusion framework for privacy-preserving human activity recognition. FMCW radar robustly captures motion dynamics through range–Doppler–time signatures and remains effective under challenging visual conditions (e.g., illumination changes), while camera measurements provide complementary spatial motion cues that can help resolve ambiguities between similar activities. However, conventional camera-based HAR systems often rely on high-resolution imagery and thus raise privacy concerns, whereas radar-only HAR can struggle to reliably discriminate fine-grained activities with similar motion profiles.

To address these limitations, we used a ultra-low-resolution (4×5) camera stream and applied frame-to-frame differencing, so that the representation retains primarily motion-change information while suppressing appearance and background details. Radar measurements were represented as Doppler-based 3D cubes and unfolded into temporal sequences, which were encoded using an Transformer-based radar encoder. The modality-specific feature vectors were then fused by concatenation and classified through lightweight fully connected layers, resulting in a simple yet effective fusion architecture.

Experimental results demonstrated that the proposed fusion model consistently outperformed the corresponding single-modality baselines and achieved 98.74% overall accuracy. The fusion model improved recognition for both low-dynamics activities and dynamic activities involving pronounced temporal variations (e.g., Running and Entering). Confusion-matrix results further indicated reduced class confusions among visually or kinematically similar activities that were frequently misclassified by single-sensor models.

In terms of efficiency, the proposed model requires only approximately 11 MFLOPs, supporting real-time deployment on resource-constrained edge devices. Overall, this work presents a practical HAR solution that jointly targets privacy preservation, high recognition performance, and low computational cost, with potential applications in smart homes, indoor monitoring, and healthcare.

## Figures and Tables

**Table 1:** Radar Sensor Configuration for Data Acquisition.

Parameters	Value
Sweep Bandwidth	1.7 GHz
Center Frequency	60.75 GHz
Num Chirps/Frame	32
Num Samples/Chirp	128
Sample Rate	1 MHz
Sampling Rate (PRF)	3.997 kHz
TX/RX Antennas	TX = 1, RX = 3

**Table 2:** Dataset acquisition settings for the 15 activity classes.

Activities	Subject Distance	Viewing Angle
*Answer-Phone*, *Drinking*, *Takeoff-Glasses*	1.5 m: 125 2 m: 325	0°, 50°, 100°
*Grabbing-Handle*	1.5 m: 300 2 m: 150	0°, 30°, 70°, 100°
*Sitting*, *Standing*, *Pickup*, *Handshake*, *None*	1.5–2 m: 150 2–2.5 m: 150 2.5–3 m: 150	moved in 10° increments (0°–100°)
*Fall*, *Recovery*	1.5–2 m: 150 2–2.5 m: 150 2.5–3 m: 150	0°, 25°, 50°, 75°, 100°
*Walking*, *Running*	1.5–2 m: 150 2–2.5 m: 150 2.5–3 m: 150	longitudinal/lateral/ diagonal

**Table 3:** Layer-wise architecture and data dimension changes of the radar Transformer encoder.

Type	Operation/Kernel	Output Shape
Input	Radar cube (R,D,T)	(32,64,32)
Reshape	(T,D,R)	(32,64,32)
Flatten	64×32→2048	(32,2048)
LayerNorm + Linear	2048→16	(32,16)
CLS Token	Concatenation → length =33	(33,16)
Positional embedding	Add	(33,16)
**Transformer encoder**	Depth =1, Heads =2	(33,16)
CLS output	Select Token[0]	(16) per branch
Aggregation (3 branches)	Concatenation	(48)
*Radar-only head (single-modality baseline only)*		
Fully connected	48→15	(15)

**Table 4:** Layer-wise architecture and data dimension changes of the LSTM-based radar encoder and the radar-only classification head.

Type	Operation/Kernel	Output Shape
Input	Radar cube (R,D,T)	(32,64,32)
Reshape	(T,D,R)	(32,64,32)
Flatten	64×32→2048	(32,2048)
**LSTM**	2048→16	(16) per branch
Aggregation (3 branches)	Concatenation	(48)

**Table 5:** Layer-wise architecture and data dimension changes of the radar CNN encoder.

Type	Operation/Kernel	Output Shape
Input	Radar cube (R,D,T)	(32,64,32)
Reshape	(1,R,D,T)	(1,32,64,32)
**Conv.3D**	1→8, Kernel 3×3×3, Padding 1	(8,32,64,32)
ReLU	-	(8,32,64,32)
MaxPool.3D	Downsample	(8,16,32,16)
**Conv.3D**	8→16, Kernel 3×3×3, Padding 1	(16,16,32,16)
ReLU	-	(16,16,32,16)
AdaptiveAvgPool.3D	Global pooling	(16,1,1,1)
Flatten	16×1×1×1→16	(16) per branch
Aggregation (3 branches)	Concatenation	(48)

**Table 6:** Layer-wise architecture and data dimension changes of the LSTM-based camera encoder and the camera-only classification head.

Type	Operation	Output Shape
Input	Camera cube (H,W,T)	(5,4,40)
Reshape	(T,H,W)	(40,5,4)
Flatten	5×4→20	(40,20)
**LSTM**	20→48	(48)
*Camera-only head (single-modality baseline only)*		
Fully connected	48→15	(15)

**Table 7:** Layer-wise architecture and data dimension changes of the camera CNN encoder.

Type	Operation	Output Shape
Input	Camera cube (H,W,T)	(5,4,40)
Reshape	(1,H,W,T)	(1,5,4,40)
**Conv.3D**	1→16, Kernel 3×3×3, Padding 1	(16,5,4,40)
ReLU	-	(16,5,4,40)
MaxPool.3D	Downsample	(16,2,2,20)
**Conv.3D**	16→48, Kernel 3×3×3, Padding 1	(48,2,2,20)
ReLU	-	(48,2,2,20)
AdaptiveAvgPool.3D	Global pooling	(48,1,1,1)
Flatten	48×1×1×1→48	(48)

**Table 8:** Layer-wise architecture and data dimension changes of the camera Transformer encoder.

Type	Operation	Output Shape
Input	Camera cube (H,W,T)	(5,4,40)
Reshape	(T,H,W)	(40,5,4)
Flatten	5×4→20	(40,20)
LayerNorm + Linear	20→48	(40,48)
CLS Token	Concatenation → length =41	(41,48)
Positional embedding	Add	(41,48)
**Transformer encoder**	Depth =1, Heads =2	(41,48)
CLS output	Select Token[0]	(48)

**Table 9:** Classification heads for single-modality baselines and multimodal fusion. Here, $d_{\mathrm{hid}}$ denotes the hidden dimension of the fusion MLP head.

Type	Operation	Output Shape
*Radar-only head (single-modality baseline)*		
Input	Radar feature $H_{\mathrm{rad}}$	(48)
Fully connected	48→15	(15)
*Camera-only head (single-modality baseline)*		
Input	Camera feature $H_{\mathrm{cam}}$	(48)
Fully connected	48→15	(15)
*Fusion head (multimodal model)*		
Input	Concatenation $\left[H_{\mathrm{rad}};H_{\mathrm{cam}}\right]$	(96)
MLP block(s)	(FC + ReLU + Dropout)^×K^: $96\to d_{\mathrm{hid}}$	$\left(d_{\mathrm{hid}}\right)$
Fully connected	$d_{\mathrm{hid}}\to 15$	(15)

**Table 10:** Training Environment and Key Hyperparameter Settings.

Parameters	Value
OS/Python	Windows 11/Python 3.11.5
GPU	NVIDIA GeForce RTX 4090
CUDA/PyTorch	12.4/2.5.1
Optimizer	Adam ($\beta_{1}\;=\;0.9$, $\beta_{2}\;=\;0.999$)
Initial Learning Rate	$10^{-3}$
Loss Function	Cross-Entropy Loss
Label Smoothing	0.1
Batch Size	32
Epochs	1000
LR Scheduler	ReduceLROnPlateau (monitor: val loss)
LR Reduce Factor/Patience	0.3/50 epochs
Minimum Learning Rate	$10^{-5}$
Gradient Clipping	$\ell_{2}$ norm ≤2.0
Reproducibility	Fixed seeds (Python/NumPy/PyTorch)
Deterministic Setting	cuDNN benchmark off; deterministic on
Model Selection	Best validation accuracy (weights saved)
Dataset Split	Train 80%, Test 20%

**Table 11:** Performance Comparison of Single and Fusion Models.

Model	Accuracy (%)	FLOPs (M)	Params (M)
Transformer-based Radar Single	95.26	10.74	0.314
LSTM-based Camera Single	95.63	0.554	0.014
Fusion (proposed)	**98.74**	11.01	0.350
Feng et al. [23]	98.89	25,856.53	15.20
Zhou et al. [24]	99.70	2464.35	10.53

**Table 12:** Comparison of single-modality performance with different encoder backbones. Note that only the radar input has two representation variants (Doppler cube vs. STFT-based spectrogram), while the camera input uses a fixed representation.

Sensor	Input Data	Feature Extractor	Accuracy (%)	FLOPs (M)	Params (M)
Radar	Doppler	CNN	98.52	127.80	0.004
		Transformer	95.26	10.74	0.314
		LSTM	85.78	12.71	0.397
	STFT	CNN	86.67	121.14	0.004
		Transformer	87.26	10.31	0.311
		LSTM	91.85	12.31	0.385
Camera	–	CNN	96.00	2.01	0.022
		Transformer	95.93	8.50	0.201
		LSTM	95.63	0.55	0.014

**Table 13:** Performance and computational cost of fusion models under different radar–camera encoder combinations, with radar input representations (Doppler cube vs. STFT-based spectrogram). The best accuracy and the lowest FLOPs are highlighted in bold.

Radar Encoder		Camera Encoder	Accuracy (%)	FLOPs (M)	Params (M)
Type	Input Data				
CNN	Doppler	CNN	99.26	131.08	0.093
		Transformer	98.30	137.57	0.272
		LSTM	**99.41**	129.42	0.085
Transformer		CNN	98.37	12.76	0.358
		Transformer	97.93	19.26	0.537
		LSTM	98.74	11.01	0.350
LSTM		CNN	98.67	14.74	0.441
		Transformer	96.44	20.85	0.620
		LSTM	98.67	13.28	0.433
CNN	STFT	CNN	98.59	124.44	0.093
		Transformer	98.07	130.94	0.272
		LSTM	97.63	122.76	0.085
Transformer		CNN	96.52	12.34	0.355
		Transformer	96.00	19.13	0.533
		LSTM	98.07	**10.89**	0.347
LSTM		CNN	96.22	14.34	0.429
		Transformer	96.74	20.45	0.608
		LSTM	97.85	12.89	0.421

**Table 14:** Confusion matrix of the LSTM-based Radar Single model (max = 90).

	Predict	Answer-Phone	Drinking	Takeoff-Glasses	Grabbing-Handle	Sitting	Standing	Pickup	Fall	Recovery	Handshake	None	Walking	Running	Entering	Exiting
True																
Answer-Phone		67	16	7	0	0	0	0	0	0	0	0	0	0	0	0
Drinking		10	77	3	0	0	0	0	0	0	0	0	0	0	0	0
Takeoff-Glasses		5	12	73	0	0	0	0	0	0	0	0	0	0	0	0
Grabbing-Handle		2	0	0	88	0	0	0	0	0	0	0	0	0	0	0
Sitting		0	0	0	0	81	2	0	5	0	1	1	0	0	0	0
Standing		0	0	0	0	20	38	20	0	1	11	0	0	0	0	0
Pickup		0	0	0	0	2	11	56	3	2	14	0	2	0	0	0
Fall		0	0	0	0	0	0	0	89	1	0	0	0	0	0	0
Recovery		0	0	0	0	5	5	2	2	74	1	0	1	0	0	0
Handshake		0	0	0	0	4	3	7	0	2	74	0	0	0	0	0
None		0	1	1	0	1	0	0	0	0	0	85	0	0	0	2
Walking		0	0	0	0	0	0	1	0	0	1	0	87	1	0	0
Running		0	0	0	0	0	0	0	1	0	0	0	0	89	0	0
Entering		0	0	0	0	0	0	0	0	0	0	0	0	0	90	0
Exiting		0	0	0	0	0	0	0	0	0	0	0	0	0	0	90

**Table 15:** Confusion matrix of the LSTM-based Camera Single model (max = 90).

	Predict	Answer-Phone	Drinking	Takeoff-Glasses	Grabbing-Handle	Sitting	Standing	Pickup	Fall	Recovery	Handshake	None	Walking	Running	Entering	Exiting
True																
Answer-Phone		85	2	1	2	0	0	0	0	0	0	0	0	0	0	0
Drinking		2	78	0	1	2	1	0	0	0	0	3	3	0	0	0
Takeoff-Glasses		1	0	89	0	0	0	0	0	0	0	0	0	0	0	0
Grabbing-Handle		0	0	0	89	0	0	0	0	0	0	1	0	0	0	0
Sitting		0	0	0	0	85	0	2	2	0	0	1	0	0	0	0
Standing		0	0	0	0	0	89	0	0	1	0	0	0	0	0	0
Pickup		0	0	0	0	1	0	88	0	0	0	0	1	0	0	0
Fall		0	1	0	0	2	0	0	86	0	0	0	0	1	0	0
Recovery		0	2	0	0	0	0	0	0	87	0	1	0	0	0	0
Handshake		0	0	0	0	0	0	0	0	0	85	5	0	0	0	0
None		0	2	0	0	0	0	0	0	0	0	86	2	0	0	0
Walking		0	3	0	0	0	0	2	0	0	4	1	78	2	0	0
Running		0	0	0	0	0	0	0	0	0	0	0	1	89	0	0
Entering		0	0	0	0	0	0	0	0	0	1	0	2	0	87	0
Exiting		0	0	0	0	0	0	0	0	0	0	0	0	0	0	90

**Table 16:** Confusion matrix of the LSTM-based Fusion model (max = 90).

	Predict	Answer-Phone	Drinking	Takeoff-Glasses	Grabbing-Handle	Sitting	Standing	Pickup	Fall	Recovery	Handshake	None	Walking	Running	Entering	Exiting
True																
Answer-Phone		88	2	0	0	0	0	0	0	0	0	0	0	0	0	0
Drinking		6	84	0	0	0	0	0	0	0	0	0	0	0	0	0
Takeoff-Glasses		0	0	90	0	0	0	0	0	0	0	0	0	0	0	0
Grabbing-Handle		0	0	0	90	0	0	0	0	0	0	0	0	0	0	0
Sitting		0	0	0	0	88	1	0	0	0	0	1	0	0	0	0
Standing		0	0	0	0	1	87	0	0	1	1	0	0	0	0	0
Pickup		0	0	0	0	0	0	88	0	0	2	0	0	0	0	0
Fall		0	0	0	0	0	0	0	90	0	0	0	0	0	0	0
Recovery		0	0	0	1	0	0	0	0	88	1	0	0	0	0	0
Handshake		0	0	0	0	0	0	1	0	1	87	1	0	0	0	0
None		0	0	0	0	0	0	0	0	0	0	90	0	0	0	0
Walking		0	0	0	0	0	0	1	0	0	0	0	88	1	0	0
Running		0	0	0	0	0	0	0	0	0	0	0	0	90	0	0
Entering		0	0	0	0	0	0	0	0	0	0	0	0	0	90	0
Exiting		0	0	0	0	0	0	0	0	0	0	0	0	0	0	90

**Table 17:** Misclassifications of *Standing* and *Pickup* by distance (D) and viewing angle (V) in the LSTM-based radar-only model (each cell reports the misclassified predicted class and the number of samples).

**Standing**												
	V	0°	10°	20°	30°	40°	50°	60°	70°	80°	90°	100°
D												
1.5–2 m			Sitting(3)	Sitting(1)	Handsh(2)	Sitting(1)	Sitting(1)	Pickup(3)	Handsh(1)		Handsh(1)	
							Pickup(1)	Recover(1)				
							Handsh(1)					
2–2.5 m		Sitting(1)	Sitting(1)	Pickup(1)	Pickup(3)		Sitting(2)	Sitting(4)	Sitting(1)	Pickup(1)	Sitting(1)	
			Pickup(2)		Handsh(1)		Pickup(1)		Pickup(1)		Pickup(1)	
									Handsh(1)		Handsh(1)	
2.5–3 m			Sitting(2)			Sitting(1)	Sitting(1)		Pickup(2)	Pickup(1)	Pickup(1)	
			Handsh(2)			Handsh(1)	Pickup(2)					
**Pickup**												
	V	0°	10°	20°	30°	40°	50°	60°	70°	80°	90°	100°
D												
1.5–2 m		Sitting(1)	Stand(1)	Stand(1)	Stand(1)			Stand(1)	Handsh(1)	Stand(1)		
			Handsh(1)	Fall(1)								
2–2.5 m			Stand(1)	Handsh(1)	Handsh(2)	Stand(3)	Handsh(2)	Stand(1)	Handsh(1)	Walking(2)		
			Handsh(1)		Fall(1)	Handsh(1)			Fall(1)			
			Recover(1)		Recover(1)							
2.5–3 m			Sitting(1)	Stand(1)		Handsh(2)		Handsh(2)				

**Table 18:** Comparison of radar/camera token attention ratios by activity class. *Note:* Highlighted rows indicate dynamic activities with pronounced translational motion. Overall, low-dynamics activities tend to receive higher attention on camera tokens, whereas dynamic activities tend to receive higher attention on radar tokens.

Class	Radar Token Ratio (%)	Camera Token Ratio (%)
Answer-Phone	41.31	**58.69**
Drinking	44.17	**55.83**
Takeoff-Glasses	44.00	**56.00**
Grabbing-Handle	**51.12**	48.88
Sitting	45.33	**54.67**
Standing	45.72	**54.28**
Pickup	46.65	**53.35**
Fall	49.07	**50.93**
Recovery	43.47	**56.53**
Handshake	**53.61**	46.39
None	44.93	**55.07**
Walking	50.00	50.00
Running	**71.41**	28.59
Entering	**58.42**	41.58
Exiting	**64.82**	35.18

## Data Availability

The data presented in this study are available on reasonable request from the corresponding author. The data are not publicly available due to privacy restrictions.
